# Antibiotic Perturbation and Gut Symbiont Recolonization Affect the Cold Tolerance of *Apis cerana*

**DOI:** 10.3390/ijms27209136

**Published:** 2026-10-15

**Authors:** Xun Zhang, Jie Zhao, Liankang Jiang, Chenxi Zuo, Yulong Guo, Zijun Guo

**Affiliations:** 1 State Key Laboratory of Yunnan Biological Resources Protection and Utilization, College of Plant Protection, Yunnan Agricultural University, Kunming 650201, China; 2 College of Science, Yunnan Agricultural University, Kunming 650201, China; 3 State Key Laboratory of Genetic Evolution & Animal Models, Kunming Institute of Zoology, Chinese Academy of Sciences, Kunming 650223, China

**Keywords:** *Apis cerana*, cold stress, gut symbionts, antibiotics

## Abstract

The gut microbiota contributes to host stress tolerance, yet its role in cold adaptation in the Eastern honey bee (*Apis cerana*) remains poorly understood. Here, *A. cerana* workers were treated with three antibiotics (tetracycline, streptomycin sulfate, and tylosin) at 50 μg/mL for 3 and 7 days, followed by cold survival assessment at 10 °C and 16S rRNA sequencing of the gut microbiota. Five cold tolerance-associated symbionts were subsequently evaluated in bacterial recolonization assays. Three days of antibiotic treatment significantly reduced bee survival under cold stress at 10 °C compared with the control, with the median survival time decreasing to 60 h across all groups. Seven days of streptomycin sulfate treatment further shortened the median survival time to 48 h. Antibiotics markedly depleted key symbionts, including *Apibacter* (from 23.89% to as low as 1.30%), *Snodgrassella*, and *Lactobacillus*. Recolonization with *Apilactobacillus kunkeei* and *Gilliamella apicola* significantly extended the cold median survival time to 92 h (*p* < 0.01 and *p* < 0.001, respectively), whereas *Providencia rettgeri*, *Klebsiella oxytoca*, and *Bifidobacterium indicum* extended it to 88 h. These findings indicate that gut symbionts modulate cold tolerance in *A. cerana* and that *A. kunkeei* and *G. apicola* can improve bee survival under low-temperature stress.

## 1. Introduction

Honey bees are poikilothermic insects whose physiological activities are highly dependent on ambient temperature. Temperature regulates individual development, colony behavior, and stress resistance. In temperate regions, honey bee colonies are continuously exposed to cold stress during the annual overwintering season. Cold-induced injury is a major factor limiting colony reproduction in apiculture, and the ability of bees to tolerate cold stress is defined as cold tolerance [1,2]. When ambient temperature falls below 10 °C, overwintering bees aggregate into a cluster to maintain a suitable nest temperature [3,4]. The queen resides at the cluster center. Inner worker bees generate heat through honey consumption and thoracic muscle shivering, whereas outer workers adjust cluster compactness in response to temperature fluctuations, collectively maintaining overwintering homeostasis [5]. Cold stress markedly increases the risks of mortality and pathogen infection in honey bees, with immature stages (larvae and pupae) being particularly susceptible. The cold survival of overwintering bees directly dictates colony reproductive output in the subsequent spring [6,7]. As a key immune organ, the honey bee gut plays a critical role in defending against exogenous stressors and maintaining systemic homeostasis. Gut microbiota dysbiosis can directly compromise bee physiological health [8]. Hosts and gut symbionts establish a stable mutualistic relationship: the insect gut provides a specialized colonization niche for microorganisms, while the gut microbiota regulates host growth and development, mediates nutrient metabolism, and enhances immune defense and stress adaptation [9,10,11].

Accumulating evidence indicates that gut microbiota homeostasis is tightly linked to the cold survival capacity of overwintering honey bees. Marked differences in gut microbial composition between healthy overwintering bees and collapsing colonies suggest a critical regulatory role of gut symbiotic bacteria in overwintering stress resistance [12]. The non-core genus *Bartonella* displays enhanced adaptability to overwintering dietary substrates and can provide the host with essential nutrients and key metabolic substrates, thereby supporting overwintering physiological metabolism [13]. During overwintering, core genera including *Gilliamella*, *Snodgrassella*, *Lactobacillus*, and *Bombilactobacillus* remain highly abundant, followed by non-core genera such as *Apilactobacillus* and *Bartonella.* In addition, overwintering cold stress significantly elevates the abundance of *Gilliamella*, *Bifidobacterium*, and *Lactobacillus* [14,15,16]. Gut microbiota composition exhibits species specificity among honey bees: compared with the Italian honey bee (*Apis mellifera ligustica*), the Eastern honey bee (*A. cerana*) harbors higher gut abundances of *Gilliamella* and *Apibacter* [17,18], whereas *Klebsiella oxytoca* and *Providencia rettgeri* represent the dominant taxa in overwintering *A. mellifera ligustica* colonies, with significantly increased bacterial loads [19]. Functional studies have revealed that the core symbiont *Gilliamella* degrades polysaccharides, metabolizes toxic monosaccharides, and synthesizes key lipids and pyruvate for host thermogenesis and energy storage [20,21,22]. *Snodgrassella* and *Gilliamella* co-colonize the ileum and form biofilms, synergistically maintaining the gut barrier and defending against pathogen invasion, with complementary metabolic functions [23,24]. *Apibacter* harbors genes associated with amino acid synthesis and monosaccharide detoxification, enhancing host environmental fitness [25]. Furthermore, beneficial bacteria including *Bifidobacterium*, *Bombilactobacillus*, and *Lactobacillus* participate in nutrient absorption and immune regulation. Supplementation with an *Apilactobacillus*–*Lactobacillus* consortium or *Snodgrassella* recolonization can restore bee immune function and enhance survival [26,27,28,29,30,31].

Antibiotics are commonly employed in apiculture to control bacterial diseases in honey bee larvae. However, widely used antibiotics such as tetracycline, tylosin, and penicillin–streptomycin severely disrupt the structure and abundance of the honey bee gut microbiota, inducing dysbiosis that ultimately reduces bee survival and impairs stress resistance [32]. Specifically, tetracycline treatment significantly alters gut microbial composition, reduces microbial diversity and richness, suppresses the abundance of core beneficial bacteria including *Lactobacillus*, *Snodgrassella*, and *Gilliamella*, and concurrently increases viral susceptibility in honey bees [33,34,35]. Tylosin decreases both alpha and beta diversity of the gut microbiota, causes a massive loss of beneficial bacteria in the rectum, alters the abundance structure of the ileal microbiota, and significantly accelerates bee mortality under pathogen stress [36,37,38]. Exposure to a penicillin–streptomycin mixture inhibits the expression of genes involved in nutrient metabolism and immunity, disrupts larval growth and development, and compromises systemic immune function [39]. To date, most relevant studies have addressed the effects of either cold stress or antibiotic exposure as single stressors on honey bees, whereas the response patterns and functional mechanisms of gut symbionts under the combined dual stress remain largely unexplored. In particular, the mechanisms underlying antibiotic-mediated gut microbiota dysbiosis and the consequent decline in cold tolerance under low-temperature conditions remain poorly understood.

Accumulating evidence indicates that cold stress markedly alters the gut microbiota composition of honey bees; bees can improve cold tolerance by enriching specific functional symbionts that optimize energy metabolism, detoxification capacity, and immune function. Conversely, antibiotic exposure disrupts the homeostasis of beneficial gut microbiota, exacerbating cold-induced injury and reducing overwintering survival. Strains including *Gilliamella apicola*, *Apilactobacillus kunkeei*, *Providencia rettgeri*, *Klebsiella oxytoca*, and *Bifidobacterium indicum* undergo significant abundance shifts under low-temperature conditions. Most are specifically enriched in overwintering bees and participate in host energy metabolism, detoxification, and immune regulation, thus representing potential key mediators of cold tolerance. However, current evidence only demonstrates that cold induces abundance changes in these bacteria, whereas their direct involvement in cold tolerance regulation remains functionally unvalidated. Accordingly, this study used *A. cerana* as a model to systematically investigate the effects of combined antibiotic and cold stress on bee survival and gut microbiota homeostasis and to functionally validate the roles of the five aforementioned symbionts in mediating cold tolerance. Our findings aim to advance the understanding of microbial regulatory mechanisms underlying cold stress resistance and provide a theoretical basis for overwintering management and sustainable apiculture.

## 2. Results

### 2.1. Effects of Antibiotic-Induced Gut Dysbiosis on Honey Bee Cold Survival

**Figure 1:**
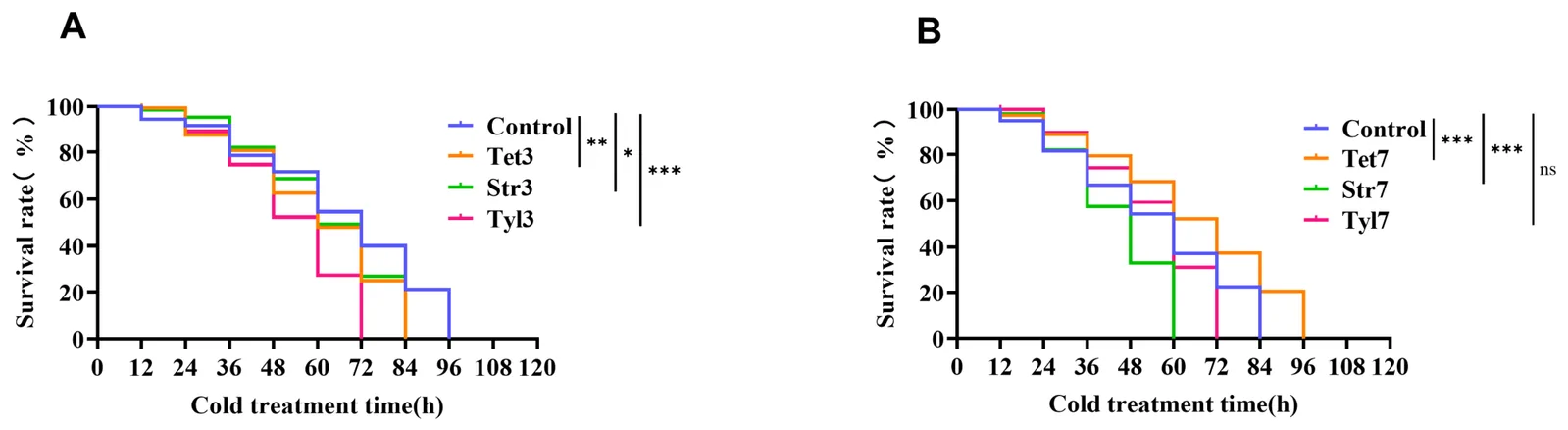
Survival analysis of honey bees under cold stress in the blank control and antibiotic treatment groups. (**A**) Survival rate analysis of bees treated with three antibiotics for 3 days. (**B**) Survival rate analysis of bees treated with three antibiotics for 7 days. * *p* < 0.05, ** *p* < 0.01, *** *p* < 0.001, ns, not significant.

In this study, honey bees were fed with three antibiotics (tetracycline, streptomycin sulfate, and tylosin) for 3 or 7 days, designated as Tet3, Str3, Tyl3, Tet7, Str7, and Tyl7, respectively. Survival analysis revealed that all three 3-day treatment groups exhibited significantly lower survival rates at 10 °C compared with the blank control (*p* < 0.01, *p* < 0.05, and *p* < 0.001, respectively), with Tyl3 exerting the strongest adverse effect on cold survival, followed by Tet3 (Figure 1A). For the 7-day treatments, Str7 significantly reduced survival at 10 °C (*p* < 0.001), whereas Tet7 significantly increased survival compared with the blank control (*p* < 0.001); no significant difference was detected between the blank control and Tyl7 (Figure 1B).

Median survival times under cold stress were estimated using the Kaplan–Meier model. In the 3-day experiment, the blank control had a median survival time of 72 h (95% CI: 65.2–78.8 h), while Tet3, Str3, and Tyl3 all showed a reduced median survival time of 60 h (95% CI: 53.8–66.3 h, 54.2–65.8 h, and 55.0–65.0 h, respectively) (Table 1). In the 7-day experiment, the median survival time of the blank control was 60 h (95% CI: 52.9–67.0 h). Tet7 extended the median survival time to 72 h (95% CI: 65.3–78.7 h), Str7 shortened it to 48 h (95% CI: 42.7–53.3 h), and Tyl7 maintained a median survival time of 60 h, identical to the blank control (95% CI: 55.5–64.5 h) (Table 2). Collectively, these results demonstrate that antibiotic exposure significantly impairs honey bee survival under cold stress, with the magnitude of the detrimental effect varying among antibiotic types.

### 2.2. Effects of Three Antibiotics on Honey Bee Gut Microbiota

**Figure 2:**
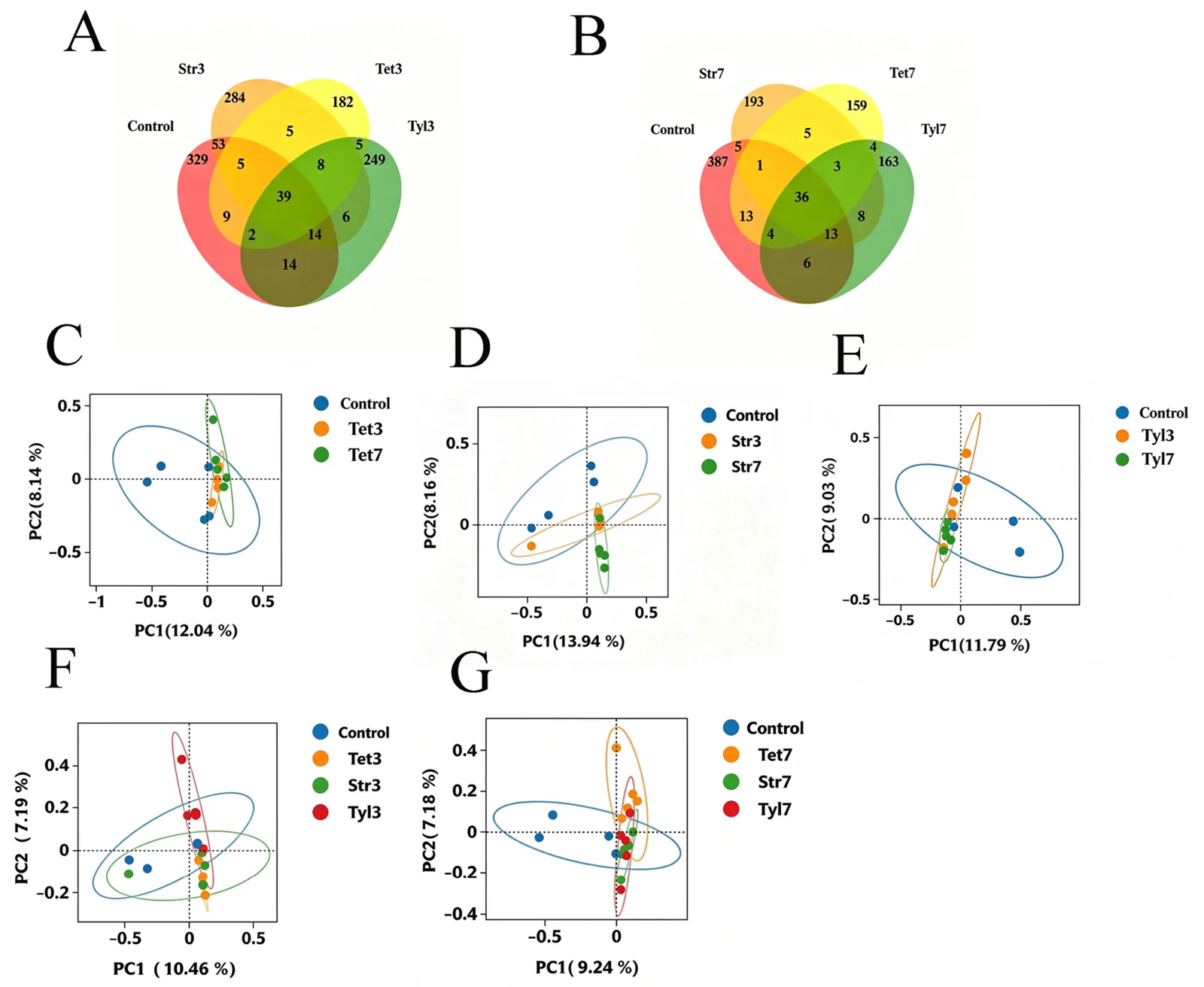
Effects of three antibiotic treatments on the diversity and community structure of the honey bee gut microbiota. (**A**,**B**) Venn diagrams showing the numbers of shared and unique OTUs among groups. The red, orange, yellow, and green circles represent the Control, streptomycin sulfate (Str), tetracycline (Tet), and tylosin (Tyl) groups, respectively. (**A**) 3-day treatment groups; (**B**) 7-day treatment groups. (**C**–**E**) Community similarity analyses showing the spatial separation of gut microbial community structure between the antibiotic treatment groups and the blank control, with confidence ellipses indicating the degree of aggregation within each group. (**F**,**G**) Community similarity analysis of the 3-day and 7-day treatment groups.

To investigate the effects of the three antibiotics on the honey bee gut microbiota, gut samples were collected and subjected to 16S rRNA sequencing. Venn diagram analysis revealed that the numbers of shared OTUs among groups were 39 and 36, respectively, and the blank control group consistently harbored more OTUs than the antibiotic treatment groups (Figure 2A,B).

In the community similarity analysis (Figure 2C–E), after 3 and 7 days of feeding with tetracycline (Tet), streptomycin sulfate (Str), and tylosin (Tyl), the confidence ellipses of all treatment groups were clearly spatially separated from that of the blank control, indicating that all three antibiotics significantly altered the gut microbial community structure of honey bees. Samples from the 3-day and 7-day groups of the same antibiotic clustered together, suggesting high similarity between microbial communities induced by different treatment durations of the same antibiotic. Notably, the Str group exhibited the most pronounced difference from the blank control, indicating that streptomycin sulfate exerted a stronger perturbing effect on gut microbiota structure than tetracycline and tylosin.

Time-point-specific analysis revealed that after 3 days of antibiotic feeding, the Tet3, Str3, and Tyl3 groups showed partial separation in microbiota distribution and were all significantly separated from the blank control, indicating differential effects of the three antibiotics after 3 days of treatment(Figure 2F). After 7 days of feeding, however, the Tet7, Str7, and Tyl7 samples clustered into a single group, suggesting high similarity among microbial communities formed by different antibiotics following 7 days of exposure(Figure 2G).

**Figure 3:**
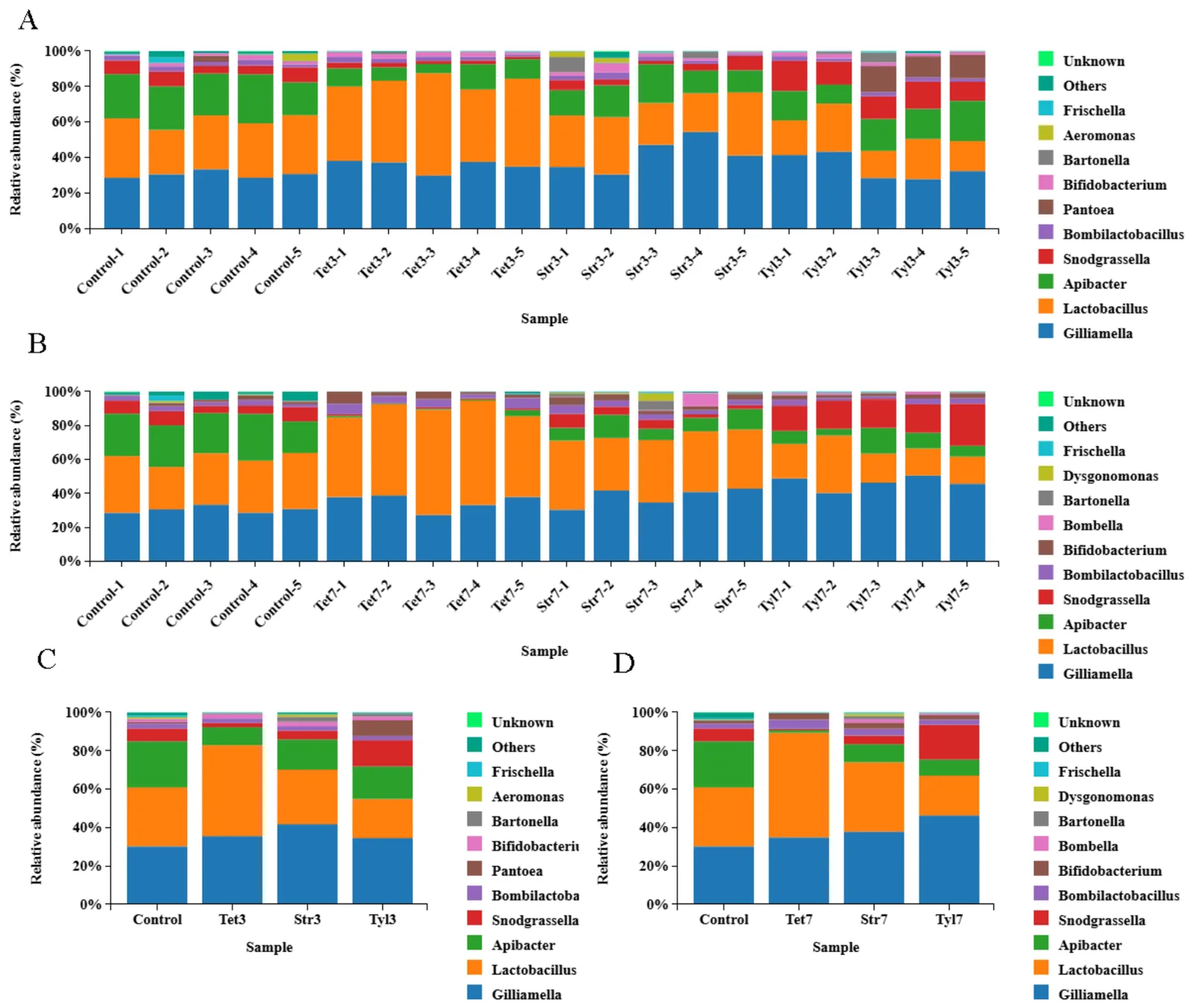
Relative abundance distribution of the honey bee gut microbiota at the genus level. (**A**,**B**) Gut microbiota composition of individual biological replicates in the 3-day and 7-day treatment groups. (**C**,**D**) Mean gut microbiota composition of the 3-day and 7-day treatment groups.

To visually illustrate the effects of the three antibiotics on the overall gut microbiota composition of honey bees, stacked bar charts of the relative abundance of intestinal microbes at the genus level were generated. The results showed that, regardless of treatment duration, the honey bee gut microbiota was dominated by *Gilliamella*, *Lactobacillus*, *Apibacter*, and *Snodgrassella*, accompanied by low-abundance genera such as *Bombilactobacillus* and *Bifidobacterium* (Figure 3A,B). In the control group, the relative abundances of the major genera were relatively evenly distributed. Following antibiotic treatment, marked shifts in microbial community structure were observed: in the tetracycline groups, the proportions of *Apibacter* and *Snodgrassella* were substantially reduced, and these changes became more pronounced with prolonged treatment. In the streptomycin sulfate groups, the proportion of *Apibacter* showed a moderate decrease. In the tylosin groups, the proportion of *Lactobacillus* decreased, while that of *Gilliamella* was relatively elevated (Figure 3C,D). These results visually demonstrate that all three antibiotics can alter the overall gut microbiota composition of honey bees to varying degrees, with distinct perturbation patterns among different antibiotics.

**Figure 4:**
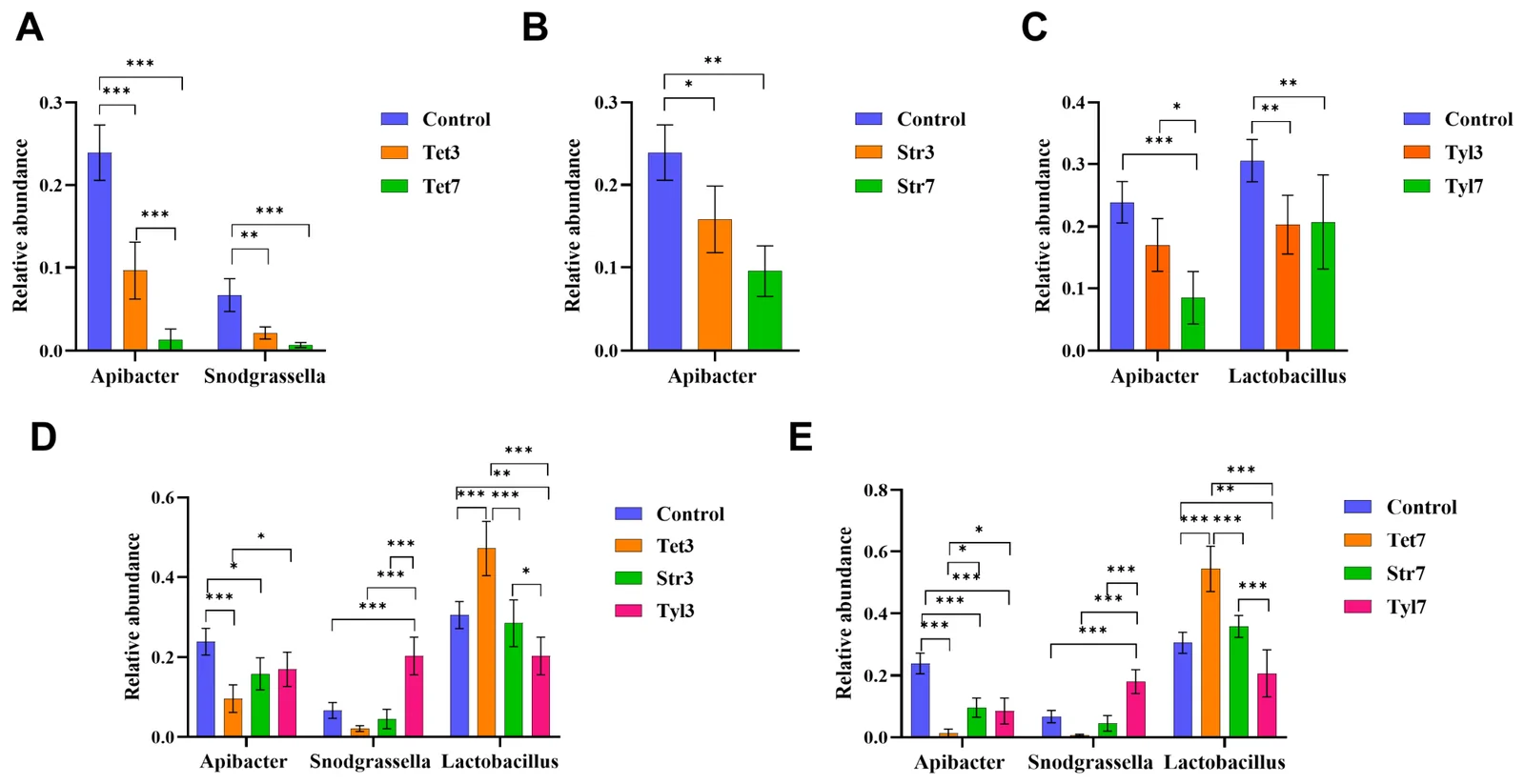
Effects of three antibiotic treatments on the relative abundance of gut bacterial genera in honey bees. (**A**) Relative abundance of *Apibacter* and *Snodgrassella* in the tetracycline treatment groups. (**B**) Relative abundance of *Apibacter* in the streptomycin sulfate treatment groups. (**C**) Relative abundance of *Apibacter* and *Lactobacillus* in the tylosin treatment groups. (**D**,**E**) Inter-group comparisons of the relative abundances of *Apibacter*, *Snodgrassella*, and *Lactobacillus* among the 3-day and 7-day treatment groups. * *p* < 0.05, ** *p* < 0.01, *** *p* < 0.001.

Following tetracycline treatment, *Apibacter* decreased from 23.89% (control) to 9.67% in Tet3 and 1.30% in Tet7 (both *p* < 0.001), with Tet7 significantly lower than Tet3 (*p* < 0.001). *Snodgrassella* decreased from 6.70% to 2.14% (*p* < 0.01) and 0.68% (*p* < 0.001), respectively (Figure 4A). Following streptomycin sulfate treatment, *Apibacter* decreased from 23.89% to 15.86% in Str3 (*p* < 0.05) and 9.60% in Str7 (*p* < 0.01) (Figure 4B). Following tylosin treatment, *Apibacter* decreased to 17.00% in Tyl3 (*p* = 0.0803, not significant) and significantly to 8.48% in Tyl7 (*p* < 0.001), with Tyl7 lower than Tyl3 (*p* < 0.05). *Lactobacillus* decreased from 30.55% (control) to 20.30% in Tyl3 and 20.68% in Tyl7 (both *p* < 0.01) (Figure 4C).

Inter-group comparisons (Figure 4D,E) showed that among the 3-day groups, *Apibacter* was higher in Tyl3 than Tet3 (*p* < 0.05), *Snodgrassella* was higher in Tyl3 than the other three groups (*p* < 0.001), and *Lactobacillus* was higher in Tet3 than the other three groups (*p* < 0.001) but lower in Tyl3 than Str3 (*p* < 0.05). Among the 7-day groups, *Apibacter* was lower in Tet7 than the other three groups (*p* < 0.001, *p* < 0.05), *Snodgrassella* was higher in Tyl7 than the other three groups (*p* < 0.001), and *Lactobacillus* was higher in Tet7 than the other three groups (*p* < 0.001) but lower in Tyl7 than Str7 (*p* < 0.001). In summary, tetracycline significantly reduced *Apibacter* and *Snodgrassella*, streptomycin sulfate and 7-day tylosin treatment reduced *Apibacter*, and tylosin reduced *Lactobacillus*, indicating that antibiotic treatment significantly alters honey bee gut microbiota composition.

### 2.3. Isolation, Purification, and Cold Tolerance Validation of Honey Bee Gut Symbionts

**Figure 5:**
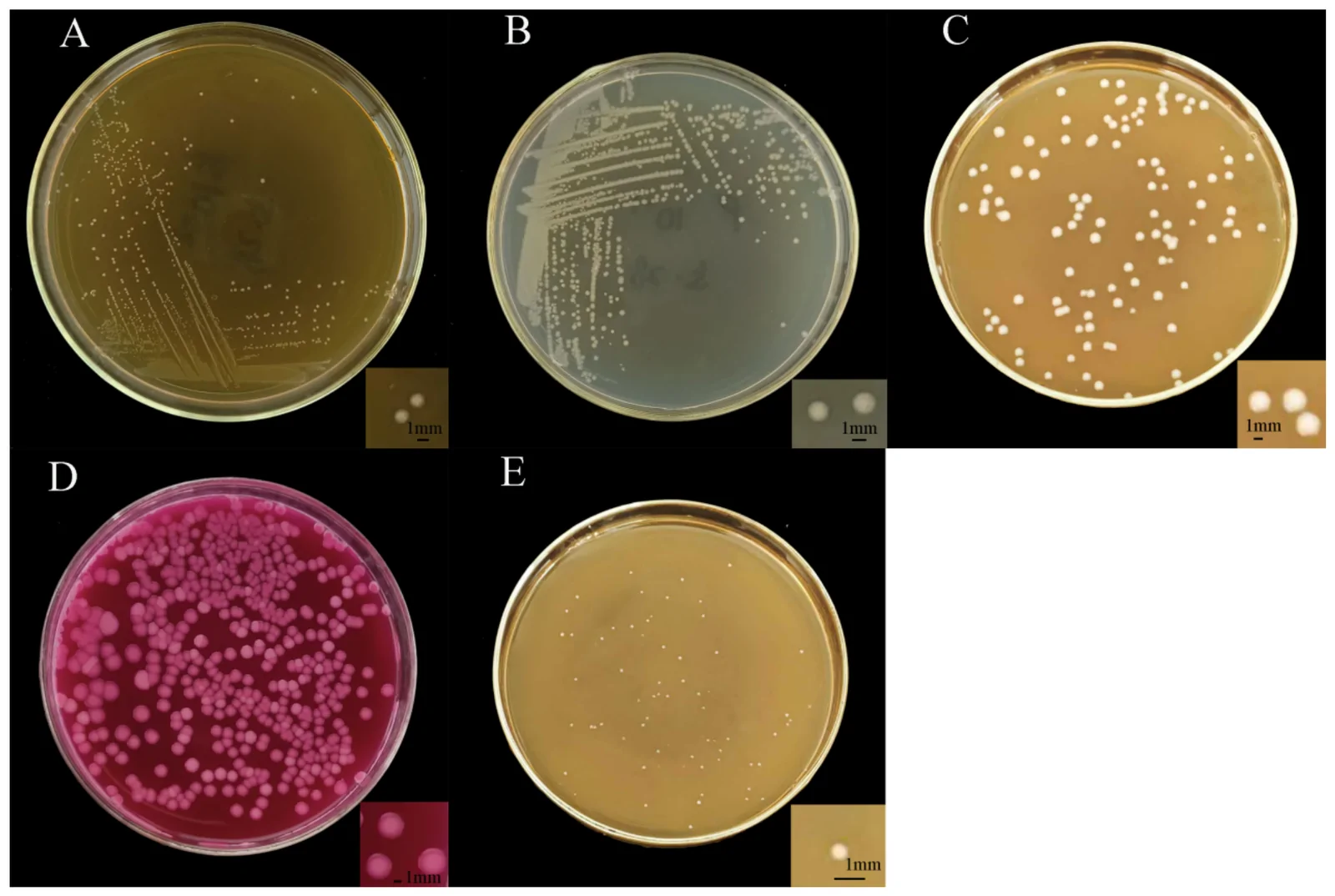
Single colony morphology of five honey bee gut symbionts. (**A**) *A. kunkeei.* (**B**) *P. rettgeri.* (**C**) *G. apicola.* (**D**) *K. oxytoca.* (**E**) *B. indicum*. Scale bars = 1 mm.

To isolate gut symbiotic bacteria from honey bees, various selective media were employed for isolation and culture, and species identification was performed via 16S rDNA sequencing. The isolation conditions and colony morphological characteristics of each strain are described below:

*A. kunkeei*: Anaerobically cultured on MRS plates supplemented with 20% fructose for 48 h. Colonies were small, milky-white and waxy, with a convex, plump appearance and a moist, viscous surface (Figure 5A).

*P. rettgeri*: Previously isolated by our research group; aerobically cultured on LB agar plates at 37 °C for 12 h. Colonies were milky white with smooth, intact margins (Figure 5B).

*G. apicola*: Aerobically cultured on MRS agar plates at 36 °C for 48 h. Colonies were grayish white, opaque, low-lying and slightly convex, and non-mucoid (Figure 5C).

*K. oxytoca*: Cultured on MacConkey–inositol–adonitol–carbenicillin (MCIAC) agar selective plates at 36 °C for 18–24 h. Colonies were purplish red, convex and plump, with a distinct precipitation ring at the periphery and a mucoid texture (Figure 5D).

*B. indicum*: Anaerobically cultured on modified MRS agar plates at 37 °C for 72 h. Colonies were milky white, circular and convex, with smooth, regular margins (Figure 5E).

**Figure 6:**
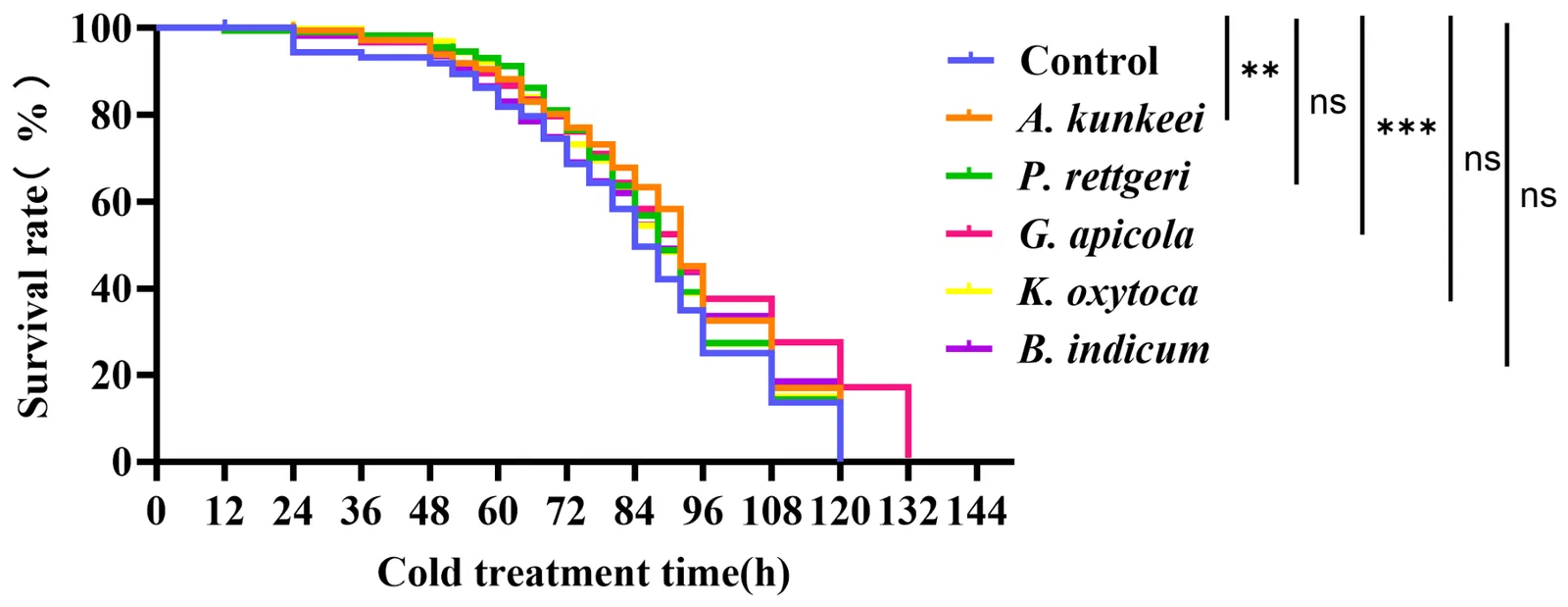
Kaplan–Meier survival curves of honey bees under cold stress in the blank control and five symbiont recolonization groups. ** *p* < 0.01, *** *p* < 0.001, ns, not significant.

Kaplan–Meier survival curves showed that, compared with the blank control, bees fed with *A. kunkeei* and *G. apicola* exhibited significantly higher survival rates under cold stress (*p* < 0.01 and *p* < 0.001, respectively), with *G. apicola* conferring the strongest cold protective effect among all tested strains. In contrast, no significant difference in survival rate was detected between the blank control and the groups fed with *P. rettgeri*, *K. oxytoca*, and *B. indicum* (*p* > 0.05) (Figure 6).

Median survival times were estimated using the Kaplan–Meier method. The blank control had a median survival time of 84 h (95% CI: 80.9–87.1 h). The groups fed with *A. kunkeei* and *G. apicola* both showed an extended median survival time of 92 h, with 95% CIs of 89.7–94.3 h and 88.3–95.7 h, respectively. The groups fed with *P. rettgeri*, *K. oxytoca*, and *B. indicum* all showed a median survival time of 88 h, with 95% CIs of 84.9–91.1 h, 84.7–91.3 h, and 83.7–92.3 h, respectively (Table 3). Collectively, *G. apicola* and *A. kunkeei* effectively improved the cold survival rate of honey bees and can serve as key functional strains for enhancing cold tolerance, whereas *P. rettgeri* and *B. indicum* showed a trend toward improved survival without reaching statistical significance.

## 3. Discussion

Low temperature threatens honey bee survival and reproduction, and successful overwintering is critical for colony persistence and spring pollination [40,41]. Gut symbionts regulate bee immunity, nutrient metabolism, and disease resistance, representing key factors for overwintering. However, cold stress alters gut microbiota composition and relative abundance, adversely affecting host health [42]. To date, studies investigating whether the gut microbiota directly regulates cold tolerance in *Apis cerana* remain scarce. Accordingly, this study established two interrelated research objectives. (1) To investigate the effects of three antibiotics administered for different durations on bee survival under cold stress, and to analyze the impacts of antibiotics on the gut microbiota using 16S rRNA sequencing. (2) To verify the regulatory roles of gut symbionts in cold stress tolerance through bacterial recolonization experiments, thereby determining whether gut symbionts directly participate in the regulation of honey bee cold tolerance.

Three-day antibiotic feeding significantly reduced the cold survival rate and median survival time of honey bees, with streptomycin sulfate exerting the strongest perturbing effect on the gut microbiota, potentially due to its broad-spectrum bactericidal activity as an aminoglycoside and its mechanism of protein synthesis inhibition. Notably, bacterial communities converged across groups after 7 days of treatment but differed after 3 days, consistent with previous reports showing that 3-day oxytetracycline and tylosin treatment significantly reduces the total gut microbial load with markedly different community structures, while prolonged antibiotic exposure induces time-dependent changes in the gut microbiota [43]. These findings suggest that community structure is co-regulated by both treatment duration and antibiotic type. Furthermore, the detrimental effect of streptomycin sulfate on cold survival was significantly greater after 7 days than after 3 days, indicating a potential time-accumulative effect of antibiotic stress, which may be associated with persistent gut microbiota disruption and residual antibiotic accumulation in bees.

The core gut genera of honey bees primarily include *Lactobacillus*, *Gilliamella*, *Apibacter*, *Snodgrassella*, *Bombilactobacillus*, and *Bifidobacterium*, consistent with previous reports. During overwintering, the relative abundances of *Bifidobacterium*, *Bombilactobacillus*, and *Lactobacillus* are significantly higher than in other seasons [44], with *Gilliamella*, *Lactobacillus*, *Bifidobacterium*, and *Snodgrassella* recognized as core functional genera, while the abundances of beneficial taxa such as *Snodgrassella*, *Acetobacteraceae*, and *Rhizobiales* fluctuate markedly during this period [45]. Interspecific comparisons revealed higher abundances of *Gilliamella* and *Apibacter* in *A. cerana* than in *A. mellifera ligustica* [13,15], whereas *K. oxytoca* and *P. rettgeri* dominate the gut microbiota of overwintering *A. mellifera ligustica* with significantly increased viable counts [16], suggesting species-specific overwintering strategies at the microbiota level.

The inhibitory effects of the three antibiotics on core genera exhibited marked species specificity: tetracycline primarily targeted *Apibacter* and *Snodgrassella*, tylosin significantly reduced *Lactobacillus*, and streptomycin sulfate exerted the most persistent inhibition on *Apibacter*. This divergence may be attributed to differences in antibiotic mechanisms of action and bacterial susceptibility profiles. The broad inhibitory effect of tetracycline on *Lactobacillus*, *Snodgrassella*, *Gilliamella*, and *Apibacter* is consistent with previous studies [33,34,35], likely because these genera are susceptible to tetracycline-mediated protein synthesis inhibition. Notably, although *Gilliamella* and *Snodgrassella* have been reported to carry several core antibiotic resistance genes [46,47,48], both were still significantly depleted by tetracycline in our study, suggesting that resistance gene carriage is insufficient to fully counteract high-dose antibiotic selection pressure. Following tylosin exposure, decreased alpha and beta diversity, substantial loss of rectal bacteria (*Lactobacillus*, *Bifidobacterium*), and increased ileal microbes (*Gilliamella*, *Snodgrassella*, *Frischella*) have been documented [36,37,38]. Such spatial redistribution may arise from differential tylosin concentrations across gut compartments and altered niche competition. Collectively, antibiotic treatment reshapes the honey bee gut microbial community by suppressing specific core genera and altering bacterial spatial distribution.

Bacterial recolonization experiments confirmed that *G. apicola* and *A. kunkeei* significantly enhanced honey bee cold survival, representing key functional strains for improving cold tolerance, whereas *P. rettgeri*, *K. oxytoca*, and *B. indicum* showed only a trend toward improved survival without reaching statistical significance. This functional divergence may be attributed to differences in metabolic profiles and the extent of their contributions under cold stress. As a core symbiont, *Gilliamella* not only metabolizes toxic monosaccharides and polyunsaturated fatty acids from pollen, but also efficiently degrades β-glucan to produce glucose and generates pyruvate through the degradation of glucuronic and galacturonic acids, thereby synthesizing lipids and key energy substrates for thermogenesis [14,18], which may represent an important mechanism underlying its cold-protective effect. Furthermore, *Gilliamella* carries a thiopeptide-like biosynthetic gene cluster that significantly inhibits the pathogen *Melissococcus plutonius*, contributing to gut defense [49]. It co-colonizes the ileum with *Snodgrassella* to form biofilms, synergistically maintaining the gut barrier, defending against pathogens, and exhibiting complementary metabolic functions [19,21]. *A. kunkeei* exhibits strong adhesive capacity and produces abundant acetic acid to alleviate intestinal oxidative stress, while surviving in high-sugar environments, showing potential for development into honey bee-specific probiotic formulations in combination with other lactic acid bacteria [50]. Additionally, *A. kunkeei* stabilizes gut microbiota structure and mitigates the toxic effects of pesticides such as acetamiprid [51], suggesting protective roles under multiple stress conditions.

In contrast, although *Apibacter* carries genes involved in amino acid synthesis and monosaccharide detoxification, and shares nitrate respiration gene clusters with *Snodgrassella*—processes critical for microaerobic microbial colonization of the gut epithelium [20]—it was not included in the recolonization experiments in this study, and its direct role in cold tolerance regulation remains to be further investigated. *Bifidobacterium*, *Bombilactobacillus*, and *Lactobacillus* are recognized as important beneficial gut taxa involved in nutrient digestion, immune regulation, and pathogen resistance [22,23,24,25,31]; notably, *Lactobacillus* significantly upregulates bee defensin expression and exhibits potent inhibitory activity against the pathogen *Hafnia alvei* in vitro [26]. In our study, although the cold median survival time was extended in the *P. rettgeri*, *K. oxytoca*, and *B. indicum* recolonization groups, the survival rates did not reach statistical significance, suggesting that enhanced cold tolerance may require synergistic interactions among multiple beneficial bacteria, and that the protective effect of single strains is limited.

In summary, a close mutualistic relationship exists between the host and its gut symbionts, which collectively maintain bee survival and stress resistance under adverse conditions through synergistic actions involving nutrient metabolism, cold thermogenesis, and immune defense. This study systematically elucidated the causal link between gut microbiota dysbiosis and decreased cold tolerance under combined antibiotic–cold stress, and verified the protective effects of *G. apicola* and *A. kunkeei* as key functional strains. These findings not only enrich the microbial regulatory theory of cold stress resistance in honey bees, but also provide new insights into overwintering management and the development of specialized probiotic formulations for honey bee colonies.

## 4. Materials and Methods

### 4.1. Survival Rate and Median Survival Time Assay

Worker bees were collected from normal colonies maintained in the experimental apiary. From each colony, adult worker bees with strong activity, intact bodies, and no visible signs of disease were gently collected using forceps. The collected bees were placed in PET rearing cups (nominal volume: 700 mL; cup mouth outer diameter: 98 mm; cup base outer diameter: 61 mm; cup height: 150 mm), with 20 bees per cup. They were fed with 50% sterile sucrose solution for 24 h in a constant-temperature and humidity incubator (QHP-300BE, Changzhou Mingyuan Machinery Equipment Co., Ltd., Changzhou, China) at 30 °C and 50% relative humidity to allow acclimation and avoid stress-induced mortality that could affect subsequent experiments. The bees were then randomly divided into control and experimental groups, with 180 workers per group. The control group was fed with 50% sterile sucrose solution, while the experimental groups were fed with 50% sterile sucrose solution containing 50 μg/mL tetracycline (Shanghai Macklin Biochemical Co., Ltd., Shanghai, China), streptomycin sulfate (Macklin, Shanghai, China), or tylosin (Shanghai Yuanye Bio-Technology Co., Ltd., Shanghai, China). Each cup of 20 bees was provided with 4 mL of the respective solution. Both groups were maintained in the incubator (30 °C, 50% relative humidity) for 3 and 7 days, with the sucrose solution refreshed every 3 days.

For the symbiont recolonization experiment, newly emerged worker bees were used. The control group was fed with 50% sterile sucrose solution, while the treatment groups were fed with sucrose solution supplemented with the corresponding symbiotic bacterial suspension until the bees reached 15 days of age.

**Figure 7:**
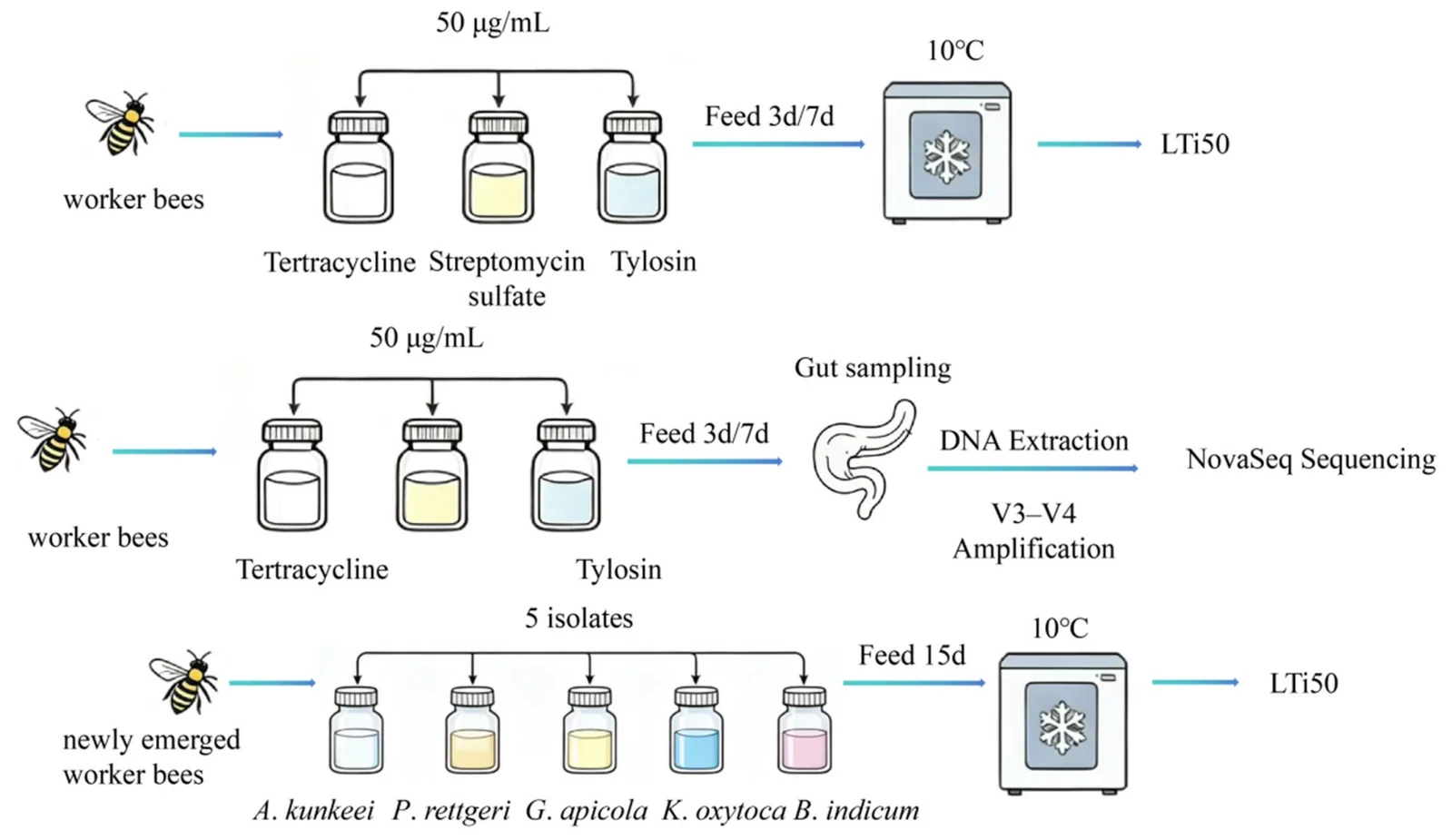
Schematic diagram of the experimental design. Worker bees of *Apis cerana* were used for antibiotic perturbation assays. Bees were fed 50 μg/mL tetracycline, streptomycin sulfate, or tylosin for 3 days and 7 days. Bees after antibiotic treatment were subjected to cold stress at 10 °C to determine the low-temperature median survival time (LTi50). Antibiotic-treated bees were dissected for gut sampling, followed by DNA extraction, V3–V4 region amplification of the 16S rRNA gene, and NovaSeq high-throughput sequencing. Five honeybee gut symbionts (*A. kunkeei*, *P. rettgeri*, *G. apicola*, *K. oxytoca*, and *B. indicum*) were isolated and cultured. Newly emerged worker bees were fed the corresponding bacterial suspensions for 15 days for gut symbiont recolonization, followed by cold treatment at 10 °C and LTi50 measurement.

For cold stress treatment, 20 workers from each group were exposed to 10 °C in a constant-temperature and -humidity incubator, and each bee was treated as an independent replicate. During the cold exposure, bees were monitored every 12 h. At each monitoring time point, the bees were transferred to 30 °C for 30 min for recovery, and the ability to fly or crawl was used as the criterion for adult survival. The number of surviving bees was recorded at each time point, and the exposure was continued until the survival rate decreased to 0%. The low-temperature median survival time (LTi50) was subsequently determined. A schematic overview of the whole experimental workflow is shown in (Figure 7).

### 4.2. 16S rRNA Sequencing

For 16S rRNA sequencing, the control group was sampled directly from the hive without laboratory rearing, while the experimental groups were collected from the hive and maintained in a constant-temperature and -humidity incubator (30 °C, 50% relative humidity), fed with 50% sterile sucrose solution containing 50 μg/mL tetracycline, streptomycin sulfate, or tylosin for 3 and 7 days, respectively. A total of 100 workers were collected per group. After cold anesthesia at 4 °C, workers were surface-sterilized in 75% ethanol for 3 min and rinsed three times with PBS. Entire guts were dissected under a laminar flow hood, pooled (16 bees per replicate, 5 biological replicates), placed in 1.5 mL tubes, snap-frozen in liquid nitrogen, and stored at −80 °C for subsequent 16S rRNA sequencing. Total DNA was extracted from the gut samples, and the V3–V4 region of the 16S rRNA gene was amplified with the primers 5′-ACTCCTACGGGAGGCAGCA-3′ (forward) and 5′-GGACTACHVGGGTWTCTAAT-3′ (reverse). Library construction and paired-end sequencing were performed on the Illumina NovaSeq 6000 platform (Illumina, San Diego, CA, USA) and the raw image data were converted into FASTQ-format sequences by base calling. Sequence denoising was performed using the DADA2 method in QIIME2 (version 2020.6) to obtain amplicon sequence variants (ASVs), and taxonomic assignment was performed against the SILVA 138 reference database using NCBI BLAST + 2.9.0 combined with the naive Bayesian classifier.

### 4.3. Isolation and Culture of Five Honey Bee Gut Symbionts

Adult worker bees were cold-anesthetized at 4 °C, surface-sterilized by immersion in 75% ethanol for 3 min, and rinsed three times with PBS. The entire gut was dissected under a laminar flow hood (VD-650, Lichen-Bangxi Instrument Technology Co., Ltd., Shanghai, China) using sterile forceps, placed in a sterile centrifuge tube containing 1 mL PBS, thoroughly homogenized, and vortexed to prepare the gut homogenate.

For the isolation of *A. kunkeei* and *B. indicum* [52], MRS (Qingdao Hopebiol Biotechnology Co., Ltd., Qingdao, China) broth supplemented with 0.5% cysteine (Beijing Solarbio Science & Technology Co., Ltd., Beijing, China) or 20% fructose ( Solarbio, Beijing, China) was used as the basal isolation medium. The gut homogenate was inoculated at 1% and 5% (*v*/*v*) and anaerobically incubated using anaerobic bags (Hopebiol, Qingdao, China) at 37 °C for 48 ± 2 h for enrichment. Subsequently, 100 μL of enriched bacterial suspension from each inoculation volume was spread onto MRS agar plates and anaerobically incubated at 37 °C for 48 ± 2 h and 72 h, respectively. *P. rettgeri* [53] was previously isolated by our research group and cultured using chromogenic solid medium (CHROMagar, Paris, France) for urinary flora localization. For the isolation of *K. oxytoca* [54] and *G. apicola* [52], the gut homogenate was serially diluted with sterile water to 10^−1^, 10^−2^,10^−3^, 10^−4^, 10^−5^, 10^−6^, and 10^−7^. Then, 100 μL of each dilution was spread onto MacConkey–inositol–adonitol–carbenicillin agar (Hopebiol, Qingdao, China) and MRS agar plates, which were incubated at 37 °C for 12–48 h.

Single colonies with typical morphology were picked and inoculated into the corresponding liquid medium, followed by incubation at 37 °C with shaking at 110 r/min for 48 h. Then, 1 mL of purified culture from each strain was collected in a sterile centrifuge tube and sent to Tianji Biotechnology Co., Ltd. (Kunming, China) for full-length 16S rDNA gene sequencing. The sequencing results were subjected to BLAST alignment against the NCBI database for species identification. The validated target strains were preserved at low temperature: a bacterial stock solution was prepared by mixing 50% sterile glycerol and bacterial suspension at a volume ratio of 1:1.5, thoroughly mixed, and stored at −80 °C.

### 4.4. Strain Resuscitation and Bacterial Suspension Preparation

The target symbiotic strains were resuscitated and activated as follows: under a laminar flow hood, a loopful of frozen bacterial stock was inoculated—*A. kunkeei*, *B. indicum*, and *G. apicola* into MRS liquid medium, and *P. rettgeri* and *K. oxytoca* into LB liquid medium (Sangon Biotech (Shanghai) Co., Ltd., Shanghai, China)—and incubated at 37 °C with shaking at 110 r/min (THZ-98A, Shanghai Yiheng Scientific Instrument Co., Ltd., Shanghai, China) for 12–48 h. Subsequently, a loopful of bacterial suspension was streaked onto the corresponding MRS or LB agar plates and incubated at 37 °C for 48 h to obtain single colonies. Single colonies were picked and inoculated into 100 mL of the corresponding liquid medium and cultured until the OD_600_ reached approximately 1.0 (Tnano-700, Tuoh Electromechanical Technology (Shanghai) Co., Ltd., Shanghai, China). For each strain, 1 mL aliquots of bacterial suspension were transferred to 1.5 mL centrifuge tubes and centrifuged (BHR-16MR, Biobase Biodustry (Shandong) Co., Ltd., Jinan, China) at 12,000 r/min for 2 min. The supernatant was discarded, and the bacterial pellet was collected. For each strain, three tubes of bacterial pellets were resuspended in 200 mL of 50% sterile sucrose solution to prepare the corresponding symbiont-containing sucrose solution. The bacterial concentration of each suspension was determined by the standard plate count method. Briefly, the bacterial suspension was serially diluted tenfold, and 100 μL of each appropriate dilution was spread onto the corresponding agar plates, which were incubated under suitable conditions until colonies appeared. Plates containing 30–300 colonies were selected for counting. The concentration was calculated using the formula: CFU/mL = (number of colonies)/(volume plated (mL) × dilution factor). The measured concentration of the bacterial suspension was 1.893 × 10^7^ CFU/mL. Each cup containing 20 bees was provided with 4 mL of the symbiont-containing sucrose solution for ad libitum consumption, corresponding to approximately 7.6 × 10^7^ CFU per cup (approximately 3.8 × 10^6^ CFU per bee).

### 4.5. Statistical Analysis

Survival rate data were analyzed using Kaplan–Meier survival analysis, and the log-rank (Mantel–Cox) test was used to compare significant differences in bee survival rates among experimental groups. Survival curves were generated using GraphPad Prism 10. Two-way analysis of variance (ANOVA) followed by Tukey’s multiple comparisons test was performed, and independent-samples t-tests were used for pairwise comparisons. All statistical analyses were conducted using IBM SPSS Statistics 27. Differences were considered statistically significant at *p* < 0.05 and highly significant at *p* < 0.01.

## 5. Conclusions

Antibiotic treatment significantly reduces honey bee cold survival and alters gut microbiota structure, with streptomycin sulfate exerting the strongest perturbing effect. Three-day antibiotic exposure uniformly decreases the median survival time to 60 h, while 7-day streptomycin further shortens it to 48 h. Tetracycline exerts a time-dependent inhibitory effect on *Apibacter* and *Snodgrassella*, whereas tylosin reduces *Lactobacillus*. Notably, different antibiotics induce similar microbiota after 7 days but distinct communities after 3 days. Recolonization with *G. apicola* and *A. kunkeei* significantly extends cold median survival to 92 h, identifying them as key functional probiotic strains, while *P. rettgeri*, *K. oxytoca*, and *B. indicum* show a trend toward improved survival. This study elucidated the functional mechanisms by which gut symbionts mediate cold tolerance in honey bees. Future studies could further explore the synergistic effects of multi-strain probiotic consortia and their field application efficacy.

## Figures and Tables

**Table 1:** Median survival time of honey bees under cold stress after 3-day antibiotic treatment.

Group	Median Survival Time (h)	95%Confidence Interval
Control	72 h	65.2–78.8
Tet3	60 h	53.8–66.3
Str3	60 h	54.2–65.8
Tyl3	60 h	55.0–65.0

**Table 2:** Median survival time of honey bees under cold stress after 7-day antibiotic treatment.

Group	Median Survival Time (h)	95%Confidence Interval
Control	60 h	52.9–67.0
Tet7	72 h	65.3–78.7
Str7	48 h	42.7–53.3
Tyl7	60 h	55.5–64.5

**Table 3:** Median survival time of honey bees under cold stress following recolonization with five symbionts.

Group	Median Survival Time (h)	95% Confidence Interval
Control	84	80.9–87.1
*A. kunkeei*	92	89.7–94.3
*P. rettgeri*	88	84.9–91.1
*G. apicola*	92	88.3–95.7
*K. oxytoca*	88	84.7–91.3
*B. indicum*	88	83.7–92.3

## Data Availability

The raw instrument-generated experimental files supporting the conclusions of this study are large-volume and not deposited in a public repository. Processed aggregated data supporting the findings are presented within the main article. The complete raw datasets are archived in our laboratory local storage and can be provided upon reasonable request to the corresponding author.
